# Diagnostic challenges of early Lyme disease: Lessons from a community case series

**DOI:** 10.1186/1471-2334-9-79

**Published:** 2009-06-01

**Authors:** John Aucott, Candis Morrison, Beatriz Munoz, Peter C Rowe, Alison Schwarzwalder, Sheila K West

**Affiliations:** 1 Department of Medicine, Johns Hopkins School of Medicine, Baltimore, Maryland, USA; 2 Lyme Disease Research Foundation of Maryland, Lutherville, Maryland, USA; 3 Dana Center for Preventive Ophthalmology, Johns Hopkins School of Medicine, Baltimore, Maryland, USA; 4 Department of Pediatrics, Johns Hopkins School of Medicine, Baltimore, Maryland, USA

## Abstract

**Background:**

Lyme disease, the most common vector-borne infection in North America, is increasingly reported. When the characteristic rash, erythema migrans, is not recognized and treated, delayed manifestations of disseminated infection may occur. The accuracy of diagnosis and treatment of early Lyme disease in the community is unknown.

**Methods:**

A retrospective, consecutive case series of 165 patients presenting for possible early Lyme disease between August 1, 2002 and August 1, 2007 to a community-based Lyme referral practice in Maryland. All patients had acute symptoms of less than or equal to 12 weeks duration. Patients were categorized according to the Centers for Disease Control and Prevention criteria and data were collected on presenting history, physical findings, laboratory serology, prior diagnoses and prior treatments.

**Results:**

The majority (61%) of patients in this case series were diagnosed with early Lyme disease. Of those diagnosed with early Lyme disease, 13% did not present with erythema migrans; of those not presenting with a rash, 54% had been previously misdiagnosed. Among those with a rash, the diagnosis of erythema migrans was initially missed in 23% of patients whose rash was subsequently confirmed. Of all patients previously misdiagnosed, 41% had received initial antibiotics likely to be ineffective against Lyme disease.

**Conclusion:**

For community physicians practicing in high-risk geographic areas, the diagnosis of Lyme disease remains a challenge. Failure to recognize erythema migrans or alternatively, viral-like presentations without a rash, can lead to missed or delayed diagnosis of Lyme disease, ineffective antibiotic treatment, and the potential for late manifestations.

## Background

Lyme disease is a multisystem infection caused by the spirochete *Borrelia burgdorferi *sensu stricto in North America. With over 27,000 new cases reported yearly, Lyme disease is the most commonly reported vector-borne disease in the United States, with the number of reported cases more than doubling between 1992 and 2006 [1]. Additional studies have shown that actual cases of Lyme disease may exceed reported cases by a factor of 6 to 12 in endemic areas [2,3]. The hallmark of early disease is a localized skin infection, erythema migrans (EM), which occurs at the site of the bite of an infected tick. Accurate identification of this rash is essential to a correct diagnosis, as serology is negative in 60% of patients early in infection when initial evaluation is likely to occur [4]. The ability of clinicians to accurately diagnose EM is unclear, but both over and under diagnosis have been reported [5,6]. Additionally, some reports suggest that up to 10% of patients with acute Lyme disease do not have a rash [7]. When untreated, the initial localized cutaneous infection may then spread hematogenously to other areas of the skin, nervous system, heart and joints.

The Centers for Disease Control and Prevention (CDC) establishes clinical and serologic criteria to standardize surveillance for Lyme disease [8]; these were recently updated in 2008 (Table 1) [9]. These criteria rely heavily on accurately identifying objective findings, especially EM, which has been shown to contribute to 70% of all diagnoses [10]. However, if the EM rash is initially missed, misdiagnosed or inadequately treated, the diagnosis of Lyme disease may be significantly delayed or missed altogether [6,11]. In addition, while CDC serologic criteria were originally designed for surveillance purposes, laboratory confirmation based on these criteria has been routinely recommended for use in individual, clinical diagnosis by such authorities as the CDC and the Infectious Disease Society of America (IDSA) [12,13]. Misinterpretation of such guidelines and the potential for clinician over-reliance on serologic confirmation for diagnosis could lead to under-diagnosis in patients with a clinically significant EM rash and false negative serology [14]. Moreover, outside of research settings there is no clinically recommended test (such as PCR or *B. burgdorferi *culture) to confirm the diagnosis of early Lyme disease if EM is not present.

**Table 1:** 2008 CDC Lyme Disease Surveillance Case Definition [9]

Confirmed	a. a case of EM with a known exposure * b. a case of EM with laboratory evidence of infection and without a known exposure c. a case with at least one late manifestation that has laboratory evidence of infection
**Probable**	Any other case of physician-diagnosed Lyme disease that has laboratory evidence of infection

**Suspected**	a. a case of EM where there is no known exposure and no laboratory evidence of infection b. a case with laboratory evidence of infection but no clinical information available (e.g. a laboratory report)

* exposure is defined as having been (less than or equal to 30 days before onset of EM) in wooded, brushy, or grassy areas (i.e. potential tick habitats) in a county in which Lyme disease is endemic (in which at least two confirmed cases have been acquired in the county or in which established populations of a known tick vector are infected with *B. burgdoreri*). A history of tick bite is not required.

The recently revised CDC case definition of Lyme disease now includes "probable" and "suspected" case classifications (Table 1). Viral-like presentations without a rash, in conjunction with positive serology, can now be considered part of the clinical spectrum of *B. burgdorferi *infection [15]. Such presentations did not meet prior CDC case definitions and only recently can be considered "probable Lyme disease" [8,9]. It is unknown whether this presentation is widely recognized by the general medical community [12].

Accurate diagnosis of early Lyme disease is important, as delayed diagnosis, missed diagnosis, or inadequate treatment with non-recommended antibiotics may have serious sequelae. A previous treatment trial with azithromycin, an antibiotic not currently recommended as first-line therapy, showed a significantly higher rate of post-treatment complications when compared with amoxicillin, a currently recommended antibiotic [16]. Further, persistent symptoms after treatment of early Lyme disease is well described and may be more common in patients with delayed diagnosis [17-19]. Estimating the incidence of post-treatment symptoms to be 5–15% and the number of new reported and unreported cases of Lyme disease to be between 160,000 and 320,000 cases a year predicts as many as 8,100 to 48,600 new patients with post treatment symptoms a year [18,20,21].

The objectives of this retrospective review are to characterize a group of community-based, ambulatory patients presenting for evaluation of possible early Lyme disease, to determine how many of these patients meet CDC criteria, and to characterize patterns of care and treatment for these patients. Few such reviews exist which address the translation of Lyme disease guidelines and recommendations to the community practice of medicine [22].

## Methods

### Patients and Setting

This review included consecutive patients presenting between August 1, 2002 and August 1, 2007 to a general internist with infectious disease training (JA) for possible early Lyme disease. Patients were either self or physician referred for consultation. The practice is located in Baltimore County, Maryland, a region characterized as high risk for Lyme disease.

### Measurements

A complete history and physical exam, including a total-body skin exam, were performed. If a skin rash meeting CDC criteria for EM was not seen, ELISA and western blot serologies for immunoglobulin M or immunoglobulin G antibodies was obtained. Extensive evaluation for possible alternative diagnoses was initiated if the patient did not have a diagnostic EM rash at the time of consultation, or if the medical history, physical exam or laboratory findings were suggestive. All subjective and objective signs and symptoms were documented on a standardized form, and any relevant medical records were reviewed. To assess neurologic involvement, documentation of cerebrospinal fluid analysis, central nervous system imaging, and nerve conduction studies was also reviewed. Patients also self-reported prior diagnoses, symptom explanations, and antibiotic and glucocorticosteroid use prior to presentation. Antibiotics considered ineffective for treatment of Lyme disease included 1^st ^generation cephalosporins, quinolones, and short-course macrolides such as 5-day azithromycin "Z-packs" or 7–10 days of clarithromycin [12].

### Definition of Cases

We included all patients referred with acute symptoms less than or equal to 12 weeks of duration. Where serologic confirmation was necessary, ELISA and Western Blots were performed by a generally available commercial lab and interpreted according to CDC criteria [23].

*Confirmed early Lyme disease *was defined by the CDC case definition [1], including either a) an EM rash or b) positive serology in the presence of an objective finding of cranial neuritis, meningitis, carditis or joint swelling. Clinical diagnosis of EM was made by a consulting physician (JA) with a high rate of specificity in EM diagnosis [24].

*Probable early Lyme disease *was defined by the recently adopted CDC case definition [9] as presentation with an acute illness, no physician-documented EM, and positive serologic confirmation, including the presence of both a positive ELISA and 2 or more bands on the IgM western blot.

*Alternative acute presentations *which did not meet criteria for early or probable early Lyme disease were further categorized into three groups.

a. Patients with specific, non-dermatologic diagnoses, including infections other than Lyme disease

b. Patients with dermatologic diagnoses

c. Patients with non-specific, viral-like syndromes without evidence of an alternative infectious process.

Case characteristics were analyzed using simple percentages and Fisher's exact tests.

This study was approved by the Institutional Review Board of the Johns Hopkins University School of Medicine and written, signed consent was obtained for all figures containing patient photographs.

## Results

**Figure 1:**
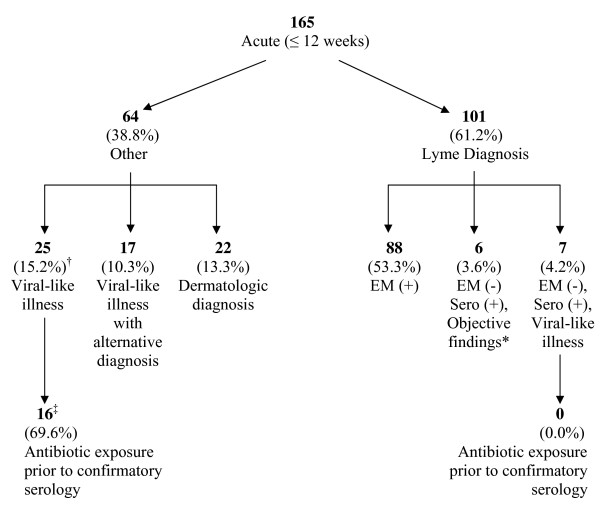
**Classification of 165 patients referred for possible acute Lyme disease**. * Presenting objective findings include: 2 patients with radiculopathy, 2 with VII nerve palsy, 1 with carditis, 1 with arthritis. ^† ^Percentages in this row represent proportion of all referred acute patients. ^‡ ^2 patients were excluded from this analysis because no confirmatory serology was drawn after antibiotics were initiated.

Of the 165 patients evaluated during the study period, 101 were diagnosed with either confirmed or probable early Lyme disease, and 64 were found to have an acute illness that either did not meet study criteria or met alternative diagnostic criteria (Figure 1). Of the 101 patients with early Lyme, 88 had an EM rash as part of their acute illness, with multiple EM lesions occurring in 13% of these patients. Of the remaining 13 seropositive patients not presenting with a diagnostic EM, 6 presented with objective neurologic or cardiac manifestations, and 7 had a viral-like illness. Thus, a total of thirteen (12.9%) of the 101 patients diagnosed with Lyme disease did not present with an EM rash.

Among the 88 patients presenting with EM, 20 (23%) had been previously misdiagnosed without the initiation of appropriate antibiotic treatment (Table 2). In 13 (65%) of these 20 patients, the rash had been observed but incorrectly identified or treated. Rashes were often misidentified by clinicians and patients as a spider bite, cellulitis, or shingles (data not shown). For the remaining 7, the EM was not discovered or was not present at the time of initial misdiagnosis. Fourteen (70%) of those patients with EM who were previously misdiagnosed with another illness had positive serology for Lyme disease.

**Table 2:** Characteristics and presenting features of previously misdiagnosed patients (n = 27)

	EM (+) n = 88	EM (-), Sero (+), Objective findings n = 6	EM (-), Sero (+), Viral-like illness n = 7	Total n = 101
Patients with prior misdiagnosis (% of all patients in category)	20 (22.7)	5 (83.3)	2 (28.6)	27 (26.7)

Sero (+) for early Lyme disease	14	5	2	21

Exposed to ineffective antibiotic prior to diagnosis	10	1	0	11

Exposed to steroids prior to diagnosis	6	2	0	8

Of the 13 seropositive patients diagnosed with Lyme disease without an EM present, 7 (53.8%) had been previously misdiagnosed (Table 2). Misdiagnosis occurred with greater frequency in patients with no EM and extracutaneous, objective manifestations (83%) than in patients with EM (23%: *p *= 0.004). Prior diagnoses given to these misdiagnosed patients included diverticulitis, acute coronary syndrome, sciatica, and lymphoma. Of all misdiagnosed cases, 11 (41%) received an antibiotic not recommended for treatment of Lyme disease and 8 (30%) were exposed to steroids.

Sixty four patients with acute symptoms did not meet criteria for confirmed or probable early Lyme disease (Figure 1). Twenty-five of these patients (39%) presented with negative serology and an acute, viral-like illness without objective findings. When prior antibiotic exposure was assessed for these patients, 70% had received antibiotics before diagnostic serology was obtained, compared with none of the sero-positive patients presenting with similar symptoms (p = 0.002). In 17 of these 64 patients (25%), an alternative diagnosis was made, including parvovirus, Ramsay Hunt syndrome or varicella zoster virus. A final group of 22 patients (36%) had rashes failing criteria for EM who received other dermatologic diagnoses, including local hypersensitivity tick bite reactions, round lesions of small size, or urticaria.

## Discussion

In light of recently revised CDC guidelines, this review highlights three major challenges continuing to face community practitioners: first, accurate diagnosis of EM, second, the identification of viral-like illnesses without a rash as possible Lyme disease, and third, appropriate antibiotic selection in community settings where Lyme disease is prevalent.

**Figure 2:**
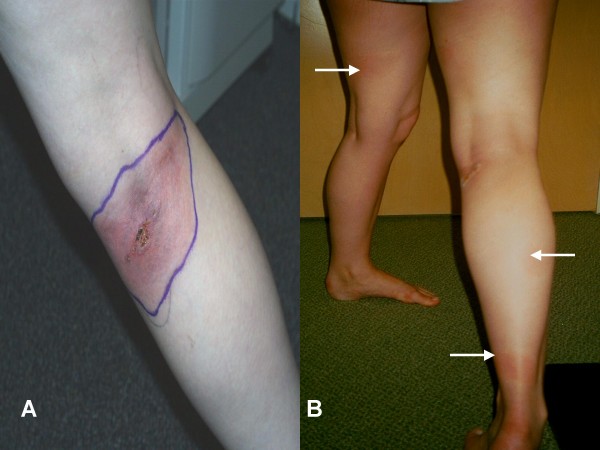
**Atypical EM with previous misdiagnosis and treatment failure**. A 26 year old woman was diagnosed with a brown recluse spider bite and treated with oral cephalexin and prednisone for an erythematous lesion with a central eschar (panel A). Ten days later, she has persistent malaise and generalized aches and was seen by one of the authors (JA). She was found to have healing of the primary EM lesion with the appearance of new secondary lesions (panel B). Serology showed ELISA reactivity with positive confirmatory IgM and IgG western blots. Treatment with oral doxycycline resulted in a prompt response and resolution of the lesions and symptoms.

**Figure 3:**
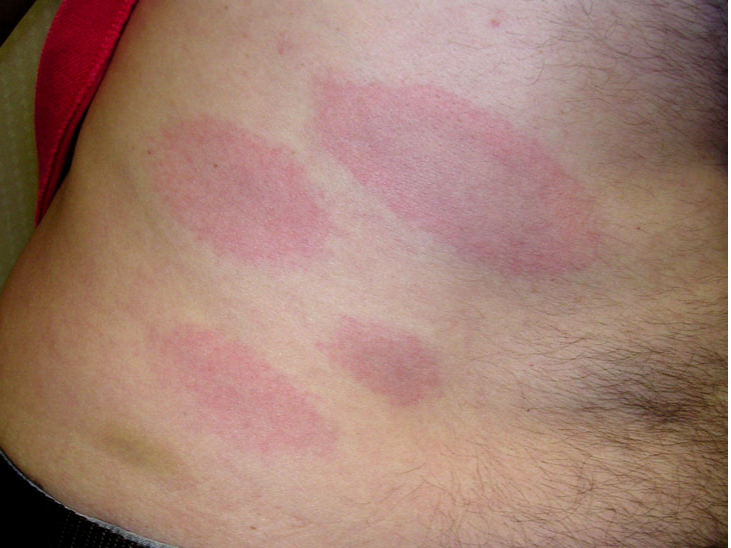
**Disseminated cutaneous EM lesions**. A 57 year old man was seen by his primary care physician for fever, fatigue, headache, and arthralgia and no specific diagnosis was made. He presented for re-evaluation to one of the authors (JA) four days later, after his wife noticed a rash. On examination, he had multiple target lesions. Serology showed ELISA reactivity with positive confirmatory IgM and IgG western blots. Treatment with oral doxycycline resulted in a prompt response and resolution of the lesions and symptoms.

**Figure 4:**
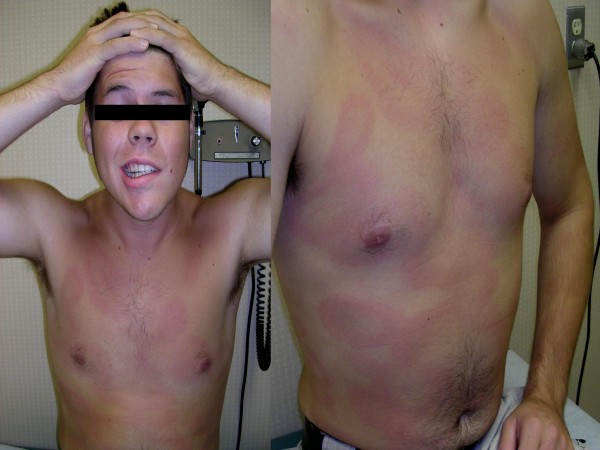
**Typical disseminated EM lesions with VII nerve palsy**. An 18 year old man was seen for new VII nerve palsy. A complete physical exam showed multiple lesions consistent with disseminated cutaneous Lyme disease. Serology for Lyme disease showed ELISA and IgM reactivity. Treatment with oral doxycycline resulted in a prompt resolution of the rash, with recovery of the VII nerve palsy over the next several weeks.

**Figure 5:**
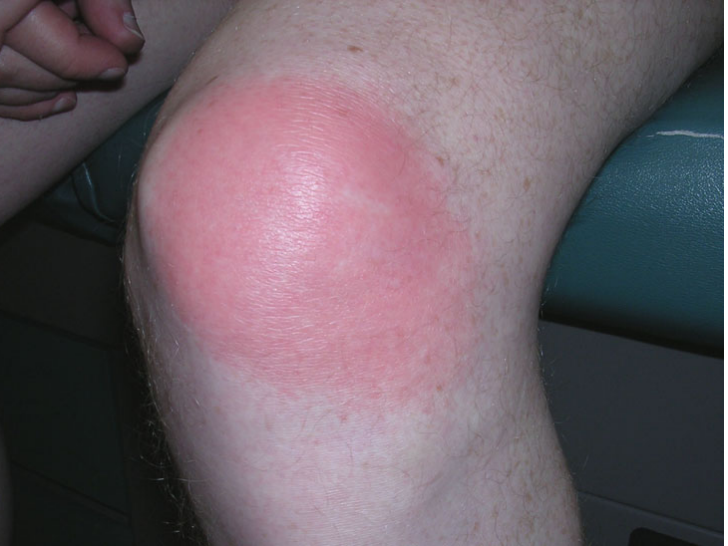
**Misdiagnosis of flu-like illness with typical, non-bull's eye EM**. A 45 year old man was seen for 'flu-like' symptoms and was diagnosed with a viral syndrome. Several days later he presented to one of the authors (JA) for re-evaluation of persistent symptoms. Complete physical exam showed a round, red, non-tender skin lesion over the later aspect of his knee. Serology for Lyme disease was negative on ELISA testing. He was treated with oral doxycycline with prompt resolution of his rash and symptoms.

**Figure 6:**
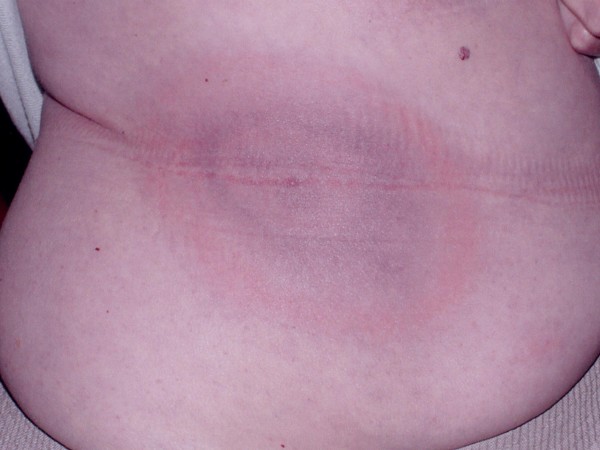
**Classic bull's eye EM with initial misdiagnosis as urinary tract infection**. A 78 year old women presented to an urgent care center with 3 days of fever, mild headache and the absence of rhinitis, cough or typical upper respiratory viral symptoms. The physical exam showed a temperature of 102 degrees Fahrenheit and a skin rash was not noted. Urinalysis showed 5–10 WBCs, a diagnosis of pylonephritis was made, and ciprofloxacin was initiated. The patient returned the following day when she noticed a large, red rash on her side. The patient was referred to one of the authors (JA) who confirmed the diagnosis of Lyme disease. Ciprofloxacin was discontinued, doxycycline initiated and the rash resolved. Serology returned with a positive ELISA and confirmatory western blot.

EM was the most common presentation of early Lyme disease in our series. However, prior misdiagnosis remained common, confirming previous reports from other endemic areas [6]. Patients and physicians often saw the EM but were unaware of its significance, understandable considering the substantial variation in its morphology [5]. While 80% of EM in the United States are uniformly red, only 19% have the stereotypical bull's eye appearance [5]. While typically circular or oval, it can also be triangular, rectangular or distorted in other ways when occurring in areas such as the neck [6]. Atypical features may include erythema with central induration, urticarial like lesions, confluent red-blue lesions mimicking ecchymosis, vesicles mimicking shingles, and central necrosis mimicking spider bites [6,25,26]. Examples of typical and atypical lesions are shown in Figures 2, 3, 4, 5 and 6. In our series, the most common misdiagnosis for EM was spider bite, consistent with observations that spider bites may be commonly over-diagnosed [27].

Even when a suspicious rash is found, there is no single, independent element in the medical history or physical exam highly sensitive for confirming the diagnosis of EM, and the specificity of the finding is unclear [5]. No confirmatory tests (such as PCR or culture) are available to community practitioners despite their potential utility in early diagnosis, and serologies offer a 60% false negative rate in the first weeks of infection [4,24,28]. Accurate diagnosis of EM requires an experienced clinical impression of the rash's appearance [5]. EM may be missed if not specifically sought on exam, as they are often asymptomatic, unseen by the patient, and found in areas such as the back, trunk and groin. In our series, several patients with an eventual EM diagnosis either received an incomplete skin exam or had a delayed EM appearance. Misidentification of EM represents a serious problem in the community, as opportunities for accurate diagnoses and appropriate treatment wane after the rash disappears. Relying on patient history or incomplete examination can thus leave the physician without a hallmark of the disease.

The common misconception that a bull's eye EM is the only diagnostic manifestation of Lyme disease continues to mislead both patients and practitioners. The absence of EM for 13% of our early Lyme cases highlights this discrepancy, with the majority of these seropositive patients (54%) developing only non-specific, viral-like illnesses without objective manifestations. Little attention has been afforded this presentation, despite the recognition that it may account for up to 9–16% of all early cases [15,29,30]. However, the IDSA guidelines for the management of Lyme disease do not specifically address nor provide treatment options for this subset of patients [12] and they are not included in treatment trials or long term outcome studies [31,32]. Our data suggests that 7% (95% CI: 2%–12%) of all acute cases may present with a viral-like illness and positive serology. Further, our identification of a subset of seronegative patients presenting with similar, non-specific viral-like illnesses of unknown etiology is notable. It is unclear whether a proportion of these patients represent probable cases who simply failed to meet current serological criteria. Until more effective antigen detection, PCR or culture become available, diagnosis of non-specific, viral-like illnesses in Lyme endemic areas will remain problematic. In high risk areas, clinicians need to be aware of such presentations in order to minimize misdiagnoses, missed treatment opportunities, and the overall population burden of Lyme disease.

Our patients with early Lyme disease defined by extracutanous, objective neurologic or cardiac disease often had systemic presentations with abdominal pain, chest symptoms or other atypical features. This misled clinicians to treat incorrect etiologies, including diverticulitis, acute coronary syndrome, sciatica, and lymphoma. Especially confusing are systemic presentations with elevated AST and ALT, previously reported in 37% of presenting patients [33,34]. Chest or abdominal pain are uncommon but elusive presentations of Lyme disease that may be due to cardiac involvement or radiculopathy involving the thoracic dermatomes [35-37].

Among previously misdiagnosed early Lyme patients, 41% received ineffective antibiotics which have been associated with treatment failures and higher relapse rates [11,12,38]. For misdiagnosed patients or those presenting with a viral-like illness, administration of ineffective antibiotics may produce unintended consequences. In studies showing suboptimal results with azithromycin, patients were often seronegative after treatment [16], raising the potential impact of sub-optimal therapy on seroconversion and further complicating reliance on a serology-based diagnosis [39]. In our series, seronegative patients presenting with a viral-like illness were significantly more likely to have been exposed to antibiotics prior to confirmatory serology than those who tested positive. The impact of ineffective antibiotics on early Lyme disease warrants further research.

The circumstances associated with steroid use in our series were a presentation of VII nerve palsy (presumably administered as treatment for idiopathic Bell's palsy) or a diagnosis of spider bite. It is unclear what additional impact the administration of steroids may have on the natural history of Lyme disease in humans [40], however steroid administration was shown to be required to achieve high rates of central nervous system infection in the Rhesus monkey model of neuroborreliosis [41].

This study has several limitations. Our inability to perform cultures or PCR on patient rashes left us without true diagnostic confirmation of the presence or absence of EM, particularly among those patients with atypical rashes. The percentage of patients with misdiagnosed rashes that showed serologic evidence of exposure to *Borrelia burgdorferi *(70%) is expected in the setting of early Lyme disease. Despite the expected level of seroreactivity, we can not be sure that the rashes we diagnosed were all due to Lyme disease. However, physicians in community practice currently do not have access to culture or PCR and must rely as we did on clinical experience when evaluating potential EM rashes. In addition, the referral population may be biased toward more complicated acute cases than are typically seen in a community population. Since patients were drawn exclusively from Maryland, results may not be generalizable to other regions where experience with Lyme diagnosis may be higher, or rates of co-infection with other tick-borne agents vary. Finally, focusing solely on those patients referred for possible acute Lyme disease fails to capture the complexity of addressing those patients referred with chronic symptoms and more complicated medical histories.

Until more accurate tests are developed for early Lyme disease, cases without a diagnostic EM rash will need to be managed carefully. In patients with cutaneous lesions where the differential diagnosis of cellulitis is not certain, empiric antibiotics should be chosen that will have activity against both Lyme disease and common agents of cellulitis [42]. In patients with viral-like illness, the clinician has the option of obtaining acute and convalescent serologies without treatment, or treating empirically with doxycycline, advantageously covering other tick-borne infections such as Rocky Mountain spotted fever or anaplasmosis [42]. Again, it should be noted that convalescent serology may be falsely negative in patients exposed to antibiotic treatment early in the course of Lyme disease [39].

## Conclusion

This case series identifies several challenges faced by practitioners in high-risk communities, challenges which may not be apparent from patients selected for treatment trials, or in non-urgent, tertiary referral centers. Diagnosing Lyme disease continues to depend on experience with accurate identification of typical and atypical EM, and confirmatory testing in early Lyme disease continues to be problematic for community clinicians. Our experience suggests that a significant percentage of cases may show alternative presentations, resulting in inaccurate diagnosis and the use of ineffective antibiotics or steroids. Despite a recent emphasis on curbing over-diagnosis of Lyme disease, these findings suggest that in non-research settings of high-risk communities, the possibility of misdiagnosis may be underestimated by the medical community. Reliance on previous CDC criteria [8] for clinical diagnosis may lead to under-diagnosis of early cases, and community clinicians should be made aware both of recent guideline changes and their limitations in individual diagnosis. Awareness of recent CDC surveillance case definition diagnostic guideline changes [9] and their limitations, awareness of alternative clinical presentations, an appreciation for the varied manifestations of EM, and the appropriate use of serologic testing will be necessary to reverse this trend.

## Competing interests

The authors declare that they have no competing interests.

## Authors' contributions

JA conceived of the study, participated in its design and coordination and helped draft the manuscript. CM participated in study conception, design, and coordination. BM participated in the study design and performed the statistical analyses. PR participated in study conception and helped draft the manuscript. AS assisted with data analysis and helped draft the manuscript. SW participated in the study design, the analyses, and helped draft the manuscript. All authors read and approved the final manuscript.

## Pre-publication history

The pre-publication history for this paper can be accessed here:

http://www.biomedcentral.com/1471-2334/9/79/prepub
